# Mojibake – The rehearsal of word fragments in verbal recall

**DOI:** 10.3389/fpsyg.2015.00350

**Published:** 2015-04-16

**Authors:** Christiane Lange-Küttner, Eva Sykorova

**Affiliations:** School of Psychology, Faculty of Life Sciences and Computing, London Metropolitan University, LondonUK

**Keywords:** word fragments, word rehearsal, working memory, visual cache, inner scribe, word form, orthographic pattern

## Abstract

Theories of verbal rehearsal usually assume that whole words are being rehearsed. However, words consist of letter sequences, or syllables, or word onset-vowel-coda, amongst many other conceptualizations of word structure. A more general term is the ‘grain size’ of word units (Ziegler and Goswami, 2005). In the current study, a new method measured the quantitative percentage of correctly remembered word structure. The amount of letters in the correct letter sequence as per cent of word length was calculated, disregarding missing or added letters. A forced rehearsal was tested by repeating each memory list four times. We tested low frequency (LF) English words versus geographical (UK) town names to control for content. We also tested unfamiliar international (INT) non-words and names of international (INT) European towns to control for familiarity. An immediate versus distributed repetition was tested with a between-subject design. Participants responded with word fragments in their written recall especially when they had to remember unfamiliar words. While memory of whole words was sensitive to content, presentation distribution and individual sex and language differences, recall of word fragments was not. There was no trade-off between memory of word fragments with whole word recall during the repetition, instead also word fragments significantly increased. Moreover, while whole word responses correlated with each other during repetition, and word fragment responses correlated with each other during repetition, these two types of word recall responses were not correlated with each other. Thus there may be a lower layer consisting of free, sparse word fragments and an upper layer that consists of language-specific, orthographically and semantically constrained words.

## Introduction

Repetition is one of the most interesting phenomena because it captures the transition from the first strenuous effort at solving a task to an automatized and much more effortless process (Logan, 1990; Fecteau and Munoz, 2003). In verbal memory, rehearsal develops at about 7 years as indicated by the onset of the phonological similarity effect at this age (Jarrold and Tam, 2011, p. 186), yet these authors hold that the onset of verbal rehearsal in general may nevertheless be gradual rather than discrete. Indeed, in the development of reading, repetition was shown to be of major importance already at a young age (Horst et al., 2011; Horst, 2013). The effect of repetition is also extensively exploited in supervised neural networks where in each repetition an error feedback signal is considered in order to optimize learning (McLeod et al., 1998). The adaptation of the neural structure often takes many sweeps. To take time to memorize to perfection by rote learning was already measured in 1885 (Ebbinghaus, 1964). Ebbinghaus meticulously recorded the time it took him to learn non-sense syllables perfectly by heart and found that on each repetition, he needed less time to achieve the same performance level. Furthermore, a neural network simulation using the original Ebbinghaus stimuli as input showed that the network learned better and more accurately without transformational (conceptual) hidden nodes, but produced the same output as input with a direct *mapping* approach. Networks always needed 200 sweeps independently whether a graphic or a phonological code was used, or homogeneous or mixed lists had to be learned – it just queued the stimuli into a sequence for output (Lange-Küttner, 2011; see also Mitchell and Zipser, 2003).

This early Ebbinghaus experiment showed that we do not necessarily need rehearse just whole words. The current study investigates whether rehearsal in a verbal recall task may actually involve word fragments. This hypothesis is backed up by recent work that shows that word structure is relevant for reading (Lange-Küttner, 2005) as well as for word memory (Lange-Küttner and Krappmann, 2011). In neural networks and reading research, usually the word onset, vowel and coda (Plaut et al., 1996) or the ‘grain size’ of units (Ziegler and Goswami, 2005) are distinguished as building blocks of a word. In memory research, participants could visually recognize word fragments that they had seen in a presentation – even if they were unable to complete the word fragment into a whole word (Challis and Sidhu, 1993; Nyberg et al., 1994; Cleary and Greene, 2000). Meaningful fragments (Cleary, 2002) and more frequent fragments (Cleary and Greene, 2001) were easier to recognize. Already 5-year-old British children who are beginning to read are able to recognize word fragments such as ‘bzn’ for the word ‘basin’ and they can even distinguish it from another fragment where instead of the phonetic cue ‘z’ for the word ‘basin’ a control cue ‘f’ is used (Rack et al., 1994). In the current study, only whole words and pseudo-words varying in familiarity and content were presented. Instead, we analyzed whether participants generated word fragments when writing down their responses in the recall phase of the word memory experiment. We used a new method that measured the percentage of correctly remembered word structure. From participants’ written recall of words, we scored not only the correctly sequenced words, but also the amount of letters in a correct sequence in fragments of a proper word. We disregarded missing or added letters, and just computed the number of correctly recalled letters as the percentage of the actual word length, because the word length effect is one of the most robust effects in word memory (Baddeley et al., 1975).

More specifically, we hypothesized that like young infants who gradually learn the correct pronunciation of a word in their spoken word production and simultaneously drop their approximations and inventions (Dromi, 1987), the young adults in the current study would be able to gradually write the correct orthography of a memorized word in their written word production in a word recall task when trying several times. All participants repeated the recall of the word lists four times, because we know that rehearsal and repetition enhances word memory as such as well as the length of a word that can be remembered (Samuels et al., 1979). We were interested whether verbal recall would improve more when the words were immediately repeated in the next three blocks, or whether a less forceful rehearsal with a randomly distributed encounter of each word list would facilitate word recall more. The distinction of massed versus distributed practice in verbal learning usually refers to the length of the inter-stimulus interval (ISI). Underwood (1961) claimed that a long ISI allows time for the successive extinction of errors, while a short ISI would suppress errors rather than extinguish them.

We did not vary the length of the ISI of trials thus each block had the same length. However, we did vary the sequence of the blocks in order to test massed versus distributed practice. One group of participants experienced each list four times in immediate succession in a kind of forced rehearsal. The other group of participants also experienced each word list four times, but the repeated word lists were presented in a mixed sequence randomized by the computer program for more incidental learning. Our prediction was that immediate repetition of a word list would support verbal recall more than a randomly distributed repetition. We assumed that an immediate repetition would also have a stronger effect because it resembles the spontaneous rehearsal of children and adults when they try to keep words in the mind for fast and safe retrieval.

Fast word learning (word mapping) in children is also dependent on semantic factors (Horst and Samuelson, 2008; Carey, 2010). Because we tested mainly young people with different ethnic backgrounds and from many countries who often spoke more languages than just English, we also monitored the content of the words. We tested names of British and international (INT) European towns (no capitals and controlled for town size). We expected that the UK towns would be easier to remember than the INT towns because of a geocentric memory bias (Baddeley, 1999, pp. 158–159). Furthermore, we tested low frequency (LF) English words against INT non-words to control familiarity. INT non-words were previously used for a cross-cultural comparison of word reading in young adults (Paulesu et al., 2000) and vocabulary learning in children (Morra and Camba, 2009) in order to avoid that non-words would vary in familiarity like existing lexical items (Treiman et al., 1990). We created the INT non-words by translating the English LF words into German, Danish, French, Italian, and Spanish. We then randomly took two or three word fragments (depending on the length of the original word) and combined them into a new word which contained legal letter sequences from the foreign words.

In summary, we designed a word recall task that controlled for repetition intensity, content and familiarity. The theoretically relevant idea for this investigation into visual word memory is to evaluate the memory fragments that the young people recalled instead of only the absolutely correct whole words that were remembered. In this way, we may be able to discover whether visual word memory rehearsal also involves word fragments, and whether these remembered fragments are a gradual approximation toward memorizing whole words. Similar response evaluations that distinguished between partially correct and completely correct responses were conducted in research with spoken stimuli and spoken responses (Storkel et al., 2006) which will allow comparison in the Discussion.

## Materials and Methods

### Participants

There were *n* = 80 participants in this study, *n* = 37 monolinguals (20 females) and *n* = 43 bilinguals (26 females). All participants were students of the London Metropolitan University, City Campus. The mean age was 27 years (SD = 9 years, range 18–55 years). Monolinguals were native English-speakers with British or US nationality. Nationalities of the bilinguals varied widely, with 27 different nationalities.

### Material

Word frequencies, range and distribution were taken from the British National Corpus (Leech et al., 2001). All LF words had a frequency below 50. The methodology of testing with INT non-words words was adopted from Paulesu et al. (2000). The generation of the INT word list from the LF words using translations into foreign languages is presented in **Table 1**. The LF words were all nouns with relatively different translations in German, Danish, French, Italian, or Spanish. We did not use words like ‘monarchy’ that would have been nearly the same in all the translations. Word fragments used for the creation of the new INT words are set in bold in **Table 1**.

**Table 1:** Translation of the low frequency words and aggregation of word fragments (underlined) into international non-words (bold).

LF words	Freq	Range	Distribution	German	Danish	French	Italian	Spanish	International Non-Words
					**Translation for WORD LIST 1**				

Harmony	13	99	86	Har**monie**	Harmonie	**Ac**cord	Armonia	Armonia	**Acmonie**
A**ss**ault	26	98	89	Anschlag	Ans**lag**	**Co**up	Attentato	Asalto	**Cosslag**
Shower	19	95	91	Dusche	**Bru**sebad	Douche	Doccia	Du**cha**	**Brucha**
Envelope	19	98	90	Umschlag	**Oms**lag	Enve**lo**ppe	Ribaltare	So**bre**	**Omslobre**
Invention	13	96	89	**Er**findung	Opfind**else**	In**ven**tion	Invenzione	Invento	**Ervenelse**
Usage	13	95	88	Gebrauch	Brug	Em**ploi**	Uso	**U**so	**Uploi**
Promotion	37	100	91	Beförde**rung**	Befordring	Avancement	Pr**om**ozione	**Asc**enso	**Ascomrung**
Mirror	43	100	88	**Sp**iegel	Spejl	M**ir**oir	Specchio	Espe**jo**	**Spirjo**

					**Translation for WORD LIST 2**				

**Jo**ke	33	100	92	Witz	Vittighed	**Es**prit	Scherzo	Broma	**Esjo**
Conte**mpt**	13	96	88	Verachtung	Vor**agt**	**Mé**pris	Disprezzo	Desacato	**Memptagt**
Textile	13	91	90	Kleidung	**Pa**kladn**ing**	Habillement	Abito	Te**xt**il	**Paxting**
Wonder	24	98	94	Wunder	**Und**er	Miracle	Miracolo	**Mar**avilla	**Undmar**
Staircase	11	86	89	**Tre**ppe	Tra**ppe**	Escalier	**Sc**ala	Escalera	**Treppesc**
Hydrogen	12	75	78	**Was**serstoff	Brint	Hydrogène	I**dro**ge**no**	N/A	**Drowasno**
Disturbance	15	99	90	Störung	Forstyrrelse	Dérange**ment**	**Dist**urbo	**Per**turbacion	**Perdistment**
**Cott**age	40	97	91	Hütte	Hytte	Cabane	Capanna	Ca**ban**a	**Bancott**

*The methodology of international compound words was adopted from Paulesu et al. (2000). Word frequency, range and distribution was taken from Leech et al. (2001). International word fragments are set in bold.*

Four different types of word lists were used, LF familiar words versus INT non-words, and UK places versus INT places, as per **Table 2**. The LF words and INT non-words (the white area in **Table 2**) were matched for amount of letters and number of spoken syllables, and so were the UK and INT places (the gray area in **Table 2**) as far as possible because also the size of the towns in terms of number of inhabitants was controlled. No names of capital towns were used. Combined letters such as ‘st’ or ‘aa’ or ‘nn’ or ‘ei’ were counted as one letter when spoken as one sound. Consonant clusters are used as one sound in experimental studies (e.g., Page et al., 2006, p. 726) and their letter count varied on average by one or two letters per word. Although consonant clusters, such as ‘st,’ are not listed in the IPA phonetic alphabet, the phonetic voiceless alveolar sibilant consonant ∫can be joined by a tie bar if for instance merged with another sound like in ‘st’ or ‘sch’ in another language (International Phonetic Association, 2005).

**Table 2:** Word Lists: low frequency words, international non-words, UK places and INT places.

LF words	PTV	INT non-words	PTV	UK places	PTV	INT places	PTV
			**Word Lists 1**				

Harmony	28	Acmonie	25	Bristol	30	Limoges	20
Assault	27	Cosslag	25	Swansea	18	Burgos	19
Shower	10	Brucha	13	Exeter	23	Odense	15
Envelope	22	Omslobre	24	Coventry	32	Hannover	31
Invention	52	Ervenelse	37	Lancaster	39	Eindhoven	37
Usage	7	Uploi	15	Leeds	14	Kursk	19
Promotion	44	Ascomrung	23	Salisbury	31	Antwerpen	37
Mirror	22	Spirjo	11	Preston	34	Grodno	25
**Total PTV**	27		22		28		25

			**Word Lists 2**				

Joke	13	Esjo	5	Hull	13	Brest	23
Contempt	45	Memptagt	35	Brighton	35	Salzburg	23
Textile	27	Paxting	30	Belfast	30	Leipzig	15
Wonder	26	Undmar	19	Dundee	22	Bilbao	16
Staircase	25	Treppesc	26	Sheffield	19	Trondheim	32
Hydrogen	27	Drowasno	29	Aberdeen	28	Debrecen	28
Disturbance	42	Perdistment	71	Southampton	51	Saarbrucken	45
Cottage	28	Bancott	39	Glasgow	15	Trieste	33
**Total PTV**	29		32		27		27

*The LF and non-words were matched for number of spoken syllables. The UK and INT town names were matched number of spoken syllables. All words per row were matched for letter length. Combined letters such as ‘st’ or ‘aa’ or ‘ei’ (consonant or vowel clusters) were counted as one letter when spoken as one sound. The exact phonotactic value (PTV; Vitevitch and Luce, 2004) is specified besides the word and is computed per word list. The PTV is related to word length as shorter words had lower values. The PTV was not related to familiarity because INT words could have the same or lower PTVs than more familiar words.*

We also controlled phonotactic values (PTV, the sum of all phoneme probabilities per word; Vitevitch and Luce, 2004) which are specified besides each word and averaged per word list in **Table 2**. Averaged values give information about the overall ease of pronunciation of a word because difficult phoneme transitions can be ameliorated by easier phoneme transition (Coleman and Pierrehumbert, 1997). These PTVs are more commonly used in studies where words need to be articulated as part of the experimental design in order to control for the ease to pronounce a word. Ease of word pronunciation according to PTV makes overt word repetition easier and has an interactive relationship with vocabulary size in children and adults (Edwards et al., 2004; Munson et al., 2005). It also facilitates repetition level but not repetition rate in neural networks (Gupta and Tisdale, 2009).

However, the current study investigated visual word memory, that is, participants saw the words and wrote down the words without a word being said. Thus the PTV was not an experimental design factor. We also did not translate the memory items into a Klattese transcription, but entered them as correctly spelled words – as the participants encountered them in the experiment – into a Phonotactic Probability Calculator that operates on the basis of an English language word data base (Kucera and Francis, 1967). The resulting PTV was related to word length as shorter words had lower values which conforms with earlier research (Bailey and Hahn, 2001). Furthermore, PTVs were not related to familiarity as the international INT words had relatively similar PTVs to the more familiar words. Also this result is in agreement with Bailey and Hahn (2001) who emphasized that PTVs can vary more drastically between native English words than in comparison to INT words. In the current study, similarities may have occurred because the INT non-words and place names were all from West European areas.

Thus, in general, when comparing the word lists, the PTVs were relatively homogeneous. The average PTV of the four memory lists in Word Lists 1 was *M* = 25.5 (range 22–28) and in Word Lists 2 it was *M* = 28.7 (range 27–32). All memory lists in Word Lists 1 were tested before those in Word lists 2. Accordingly, block sequence was separately permutated for Word Lists 1 and Word Lists 2. The computer programming software Experimental Run Time System (ERTS; Beringer, 1994) was used to present the word lists and instruct the participants. One word was presented at a time in a randomized sequence on a DOS computer with a 15 inch screen. Each word was presented in Times Large 12 font in white on a black background for 1000 ms, with an ISI of 500 ms. The presentation of the words occurred in blocks of eight words (see **Table 2**).

When programming the experiment, the four types of word lists were blocked into two sets (see Word Lists 1 and Word Lists 2 in **Table 2**). Because each word list was repeated four times, each set had 16 uniform word list presentations. In the rehearsal condition, each word list type was immediately repeated. In contrast, in the incidental learning condition, the sequence of the four times repeated blocks A, B, C, and D was randomly and completely permutated by the ERTS within each set, rather than at fixed intervals (Page et al., 2013). For instance, memory word list A_1-4_ could be repeated at any place in the random sequence of 16 blocks (e.g., A_1_, B_1_, D_1_, C_1_, A_2_, A_3_, B_2_ …) and the maximum possible space between repetitions of block type A_1_ and A_2_ was about 12 blocks if the first block was repeated only at the end of the set (e.g., A_1_, C_1_, D_1_, B_1_, D_2_, C_2_, B_2_, C_3_, D_3_, B_3_, B_4_, C_4_, A_2_, D_4_, A_3_, A_4_).

### Procedure

Participants were tested individually in a quiet computer laboratory. The experiment was vetted and approved by the Departmental Ethics Committee. Before the start of the experiment, participants were provided with a Consent form which they signed. Afterward, they received a Debrief form for informative details about the nature of the study. They were randomly assigned to one of two experimental conditions – condition 1 (immediate block repetition, forced rehearsal) or condition 2 (program generated permutated block sequence, incidental learning condition). Participants were provided with paper notepads to write down their responses. They turned over a sheet after each word list.

Instructions were given in written form on the computer display. Participants were informed that some words made sense, while others would not, and that each word list would be repeated four times during the course of the experiment. Their task was to recall as many words as they could remember. At the end of each word list presentation they were asked to write down the words on paper in any order in which they came to mind (free recall). There was no delay after the presentation of each word list and recall time was not constrained. Participants pressed the space bar to initiate the next word list (self-paced block transitions).

### Scoring

Participants’ responses were scored twice. Firstly, we scored correctly memorized and orthographically correctly written whole words. Accuracy was computed per block in per cent correct. We also scored remembered words that were recognizably part of the memory list but consisted of word fragments with only some letters in the correct sequence. We disregarded missing (omissions) or added (intrusions) letters. For instance, one word in the UK places list was ‘Salisbury.’ In the response word ‘Sailsbry’ (which has half a word with a different meaning denoting the sails of a boat), the letter ‘i’ is in the wrong place and the letter ‘u’ is missing, but all other letters are in a correct sequence. Thus, the participant scored 7 letters out of 9 correct, and received a score of 7/9 = 77.8% accuracy for this word. In another scoring example, a participant wrote ‘Sainsbury,’ that is, also this participant made a semantic mistake, but for the whole word. The participant remembered a similarly written word that denoted a British supermarket instead of a British town. In this word, the letter ‘l’ is missing and the letter ‘n’ is a wrong letter, but all other letters are in the correct sequence, 8/9 = 88.9% correct (% correct per word). These examples show that the meaning of the associated word may only have been a memory trigger as the semantic association could be quite remote to the actual stimulus, while the important feature is the orthographic similarity with the target word. The results from the accurate and the more lenient scoring were averaged per word type list, respectively, across memory list 1 and 2.

Secondly, because the lenient scoring yielded higher accuracy scores, we computed a stricter score for correct whole words which was then subtracted from the values that were obtained with the lenient scoring. The resulting scores were the pure values for just the ‘nearly correct words’ (word fragments) which we then compared with the whole word score. The comparison allowed to test whether the effect of repetition (rehearsal) relates not only to whole words but also to word fragments. If there is a gradual approximation during rehearsal toward the correct whole word, we expected that word fragments should decrease during rehearsal/repetition and would correlate with the whole word score in the subsequent block.

## Results

The first analysis compares the two scoring methods. Recall scores were analyzed with analyses of variance with repeated measures. When the Mauchley’s Test of Sphericity was significant, the degrees of freedom were adjusted according to Huynh-Feldt. In a second analysis of variance thereafter, a fragments-only score was analyzed.

We conducted a 2 (Words/Places) × 2 (Familiarity) × 4 (Repetition) × 2 (Scoring Method) × 2 (Training) analysis of variance with repeated measures on the first four factors, and type of training as a between-subject factor. Differences due to age were partialled out using the variable ‘age in years’ as covariate. In an initial analysis, we also included the variables sex and language of the participants as between-subject factors. However, the inclusion of these individual difference factors made the analysis of variance very complex. Like Logie (2011, p. 243) predicted, the main experimental results did not change when the individual difference variables were omitted. In short, men showed a memory advantage for INT places. Bilinguals profited somewhat more from immediate repetition, while monolinguals benefited from incidental learning especially when words were unfamiliar. Because these results of individual differences did not substantially contribute to the hypothesis, the statistical details of this initial analysis are not reported.

The details of the statistical results are listed in **Table 3** and are not quoted again in the text. The main effect of training type (immediate vs. distributed repetition of blocks) was not significant as a between-subject factor showing that memory performance in general did not vary in the two training groups. The main effects of scoring, familiarity and repetition were all highly significant, *p_s_* < 0.001. As expected, participants showed better word memory when also correct letter sequences in word fragments were scored (*M* = 57.2%) rather than just whole words (*M* = 49.4%). Participants remembered the familiar LF English words and UK places (*M* = 67.8%) better than the unfamiliar words, that is the INT non-words and INT places (*M* = 38.8%). Furthermore, mere repetition nearly doubled word memory accuracy which supports the hypothesis that prescribed rehearsal is an efficient facilitator (Block 1 *M* = 38.3%, Block 2 *M* = 53.0%, Block 3 *M* = 60.1%, Block 4 *M* = 61.8%).

**Table 3:** MANOVA Table of Statistical Effects, *n* = 80, for Scoring Method (left) and Composite Scores (right) Analyses of variance with repeated measures for Repetition (four times), Familiarity (low/high) and Content (words vs. geographical places).

	Whole Word scoring vs. Letter Sequence scoring				Whole Word scores vs. Word Fragments			
Statistical Effect	df	*F*	*p*	η^2^	df	*F*	*p*	η^2^
**Within-subject effects**
Scoring	**1**	**29.042**	**0.000**	**0.274**	**1**	**128.44**	**0.000**	**0.625**
Scoring*Age	1	0.000	0.986	0.000	1	1.504	0.224	0.019
Scoring*Training	1	1.910	0.171	0.024	1	1.357	0.248	0.019
Content	1	0.085	0.771	0.001	1	2.510	0.117	0.032
Content*Age	1	3.246	0.076	0.040	1	1.146	0.288	0.015
Content*Training	1	0.002	0.961	0.000	1	0.004	0.950	0.000
Familiarity	**1**	**100.716**	**0.000**	**0.567**	**1**	**35.668**	**0.000**	**0.317**
Familiarity*Age	**1**	**9.142**	**0.003**	**0.106**	1	2.724	0.103	0.034
Familiarity*Training	**1**	**11.241**	**0.001**	**0.127**	**1**	**5.759**	**0.019**	**0.070**
Repetition	**2.50**	**78.447**	**0.000**	**0.505**	**2.50**	**67.10**	**0.000**	**0.467**
Repetition*Age	**3**	**5.914**	**0.001**	**0.071**	**3**	**4.821**	**0.003**	**0.059**
Repetition*Training	2.50	1.862	0.151	0.024	**2.50**	**3.138**	**0.035**	**0.039**
Scoring*Content	**1**	**4.266**	**0.042**	**0.052**	1	0.022	0.884	0.000
Scoring*Content*Age	1	1.114	0.295	0.014	1	3.545	0.064	0.044
Scoring*Content*Training	1	0.001	0.980	0.000	1	0.002	0.968	0.000
Scoring*Familiarity	**1**	**14.447**	**0.000**	**0.158**	**1**	**105.203**	**0.000**	**0.577**
Scoring*Familiarity*Age	1	1.858	0.177	0.124	**1**	**9.886**	**0.002**	**0.114**
Scoring*Familiarity*Training	1	0.459	0.500	0.006	**1**	**10.759**	**0.002**	**0.123**
Content*Familiarity	1	3.406	0.069	0.042	1	0.064	0.800	0.001
Content*Familiarity*Age	1	0.894	0.347	0.011	**1**	**4.518**	**0.037**	**0.055**
Content*Familiarity*Training	1	0.792	0.376	0.010	1	0.104	0.748	0.001
Scoring*Content*Familiarity	**1**	**4.728**	**0.033**	**0.058**	**1**	**5.533**	**0.021**	**0.067**
Scoring*Content*Familiarity*Age	1	3.471	0.066	0.043	1	0.087	0.769	0.001
Scoring*Content*Familiarity*Training	1	1.724	0.193	0.022	1	1.477	0.228	0.019
Scoring*Repetition	3	1.220	0.303	0.016	**3**	**60.409**	**0.000**	**0.440**
Scoring*Repetition*Age	3	0.395	0.756	0.005	**3**	**4.73**	**0.003**	**0.058**
Scoring*Repetition*Training	3	1.168	0.323	0.015	3	1.177	0.319	0.015
Content*Repetition	3	0.330	0.804	0.004	3	0.879	0.451	0.011
Content*Repetition*Age	3	0.113	0.952	0.001	3	1.185	0.316	0.015
Content*Repetition*Training	3	0.078	0.972	0.001	3	0.962	0.411	0.012
Scoring*Content*Repetition	**3**	**2.727**	**0.045**	**0.034**	3	0.971	0.407	0.012
Scoring*Content*Repetition*Age	**3**	**2.816**	**0.040**	**0.035**	3	0.638	0.592	0.008
Scoring*Content*Repetition*Training	3	1.262	0.288	0.016	3	0.125	0.945	0.002
Familiarity*Repetition	3	1.320	0.269	0.017	3	3.239	0.023	0.040
Familiarity*Repetition*Age	3	1.185	0.316	0.015	3	1.870	0.135	0.024
Familiarity*Repetition*Training	3	2.520	0.059	0.032	3	1.711	0.166	0.022
Scoring*Familiarity*Repetition	**3**	**4.076**	**0.008**	**0.050**	3	1.457	0.227	0.019
Scoring*Familiarity*Repetition*Age	3	2.593	0.053	0.033	3	1.394	0.245	0.018
Scoring*Familiarity*Repetition*Training	3	0.922	0.431	0.012	3	2.298	0.078	0.029
Content*Familiarity*Repetition	3	1.189	0.315	0.015	3	0.502	0.681	0.006
Content*Familiarity*Repetition*Age	3	1.902	0.130	0.024	**3**	**0.685**	**0.006**	**0.496**
Content*Familiarity*Repetition*Training	**3**	**3.680**	**0.013**	**0.046**	3	0.427	0.012	0.931
Scoring*Content*Familiarity*Repetition	3	1.180	0.318	0.015	3	1.504	0.214	0.019
Scoring*Content*Familiarity*Repetition*Age	3	0.976	0.405	0.013	3	2.153	0.094	0.027
Scoring*Content*Familiarity*Repetition*Training	3	0.459	0.711	0.006	3	**3.561**	**0.015**	**0.044**
**Between-subject effects**
Age	1	1.607	0.209	0.020	**1**	**0.225**	**0.019**	**1.498**
Training	1	0.671	0.415	0.009	**1**	**0.162**	**0.025**	**1.990**

*The between-subject factor is Training (immediately repeated vs. permutated block sequence). Statistical effects were controlled for age in years with a covariate. Statistical effects are set in bold when the analysis of variance yielded a significant effect.*

A content by familiarity effect interacted with (1) scoring and (2) repetition and training. LF English words (*M* = 67.9%) were remembered just as well as the UK places (*M* = 67.7%), with a difference of just 0.02%. However, the INT places (*M* = 45.6%) were less difficult to remember than the INT non-words (*M* = 32.0%). As would be expected, the more challenging INT words benefited considerably from the additional letter sequence scoring compared to the whole word scoring, that is an increase of above 10% occurred (INT non-words *M* = 37.0%/*M* = 26.9%; INT places *M* = 51.1%/*M* = 40.2%). In contrast, the UK places benefited only 6.2% (*M* = 70.8%/*M* = 64.6%) and the LF English words just 3.8% (LF English words *M* = 69.8%/*M* = 66.0%). Thus, the more difficult a word was to remember the more it benefited from the additional letter sequence scoring.

**FIGURE 1:**
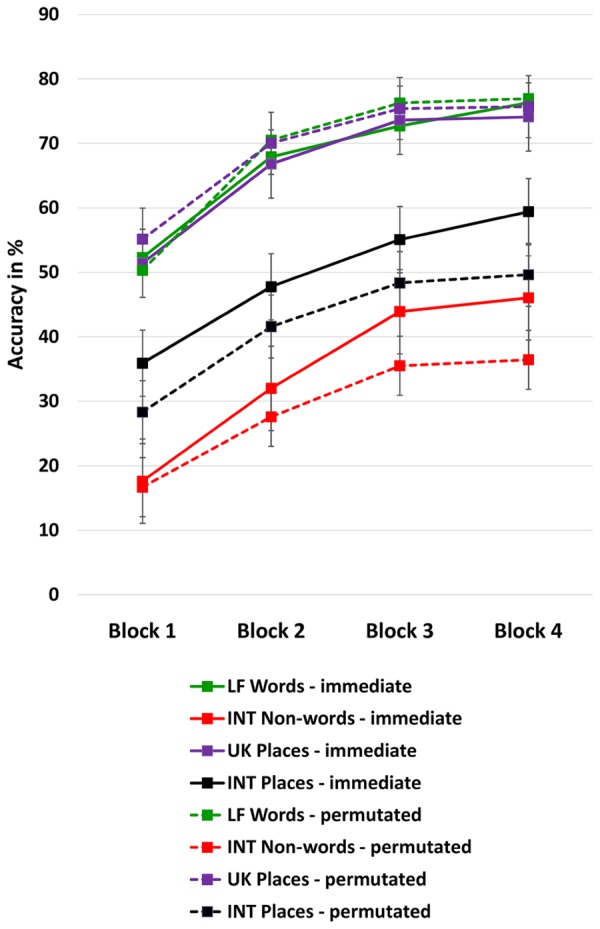
**Effect of training**. Immediate repetition is denoted by the solid lines, the permutated memory block condition is denoted by the broken line. Immediate repetition is significantly more efficient for word memory recall than incidental encounters (permutated sequence of word type lists) when words are less familiar. LF = low frequency, INT = international, UK = United Kingdom. Means are controlled for age. The bars represent the standard error.

Moreover, immediate repetition could be more efficient for word memory recall than more incidental encounters, see **Figure 1**. *Post hoc* independent *t*-tests (two-tailed) comparing the two rehearsal conditions per block showed that the immediate repetition advantage only gradually emerged for the INT words, that is, for INT places [Block 1 *t*(78) = 1.93, *p* = 0.058; Block 2 *t*(78) = 1.56, *p* = 0.123; Block 3 *t*(78) = 1.43; *p* = 0.157; Block 4 *t*(78) = 2.04, *p* = 0.044] and especially for the INT non-words [Block 1 *t*(78) = 0.543, *p* = 0.058; Block 2 *t*(78) = 1.44, *p* = 0.153; Block 3 *t*(78) = 2.74; *p* = 0.008; Block 4 *t*(78) = 3.03, *p* = 0.003].

**FIGURE 2:**
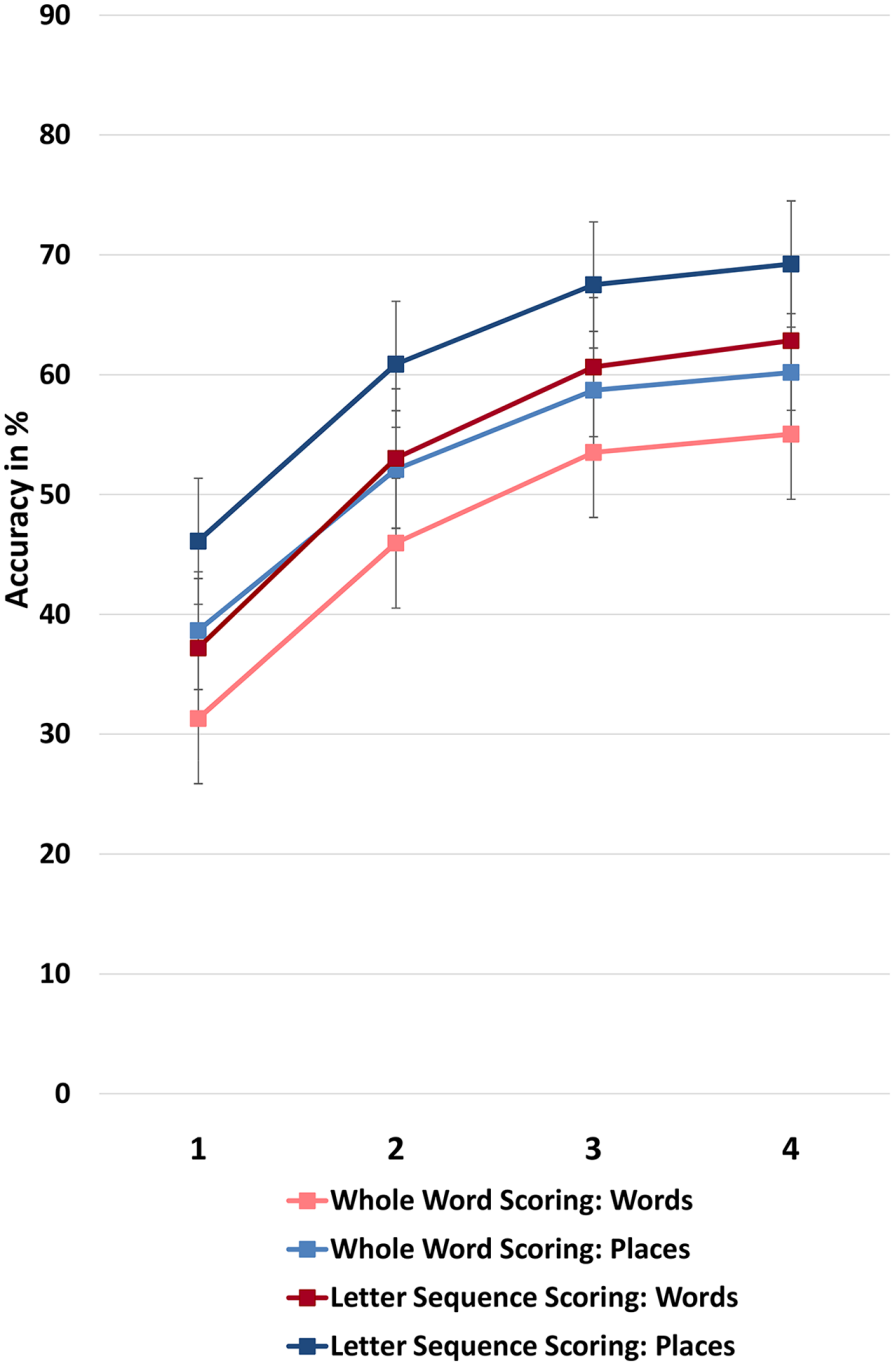
**Effects of content**. Memory for geographic place names was better than for LF words and non-words. This difference was somewhat less pronounced for completely correctly spelled words (pastel colored lines). It stayed significant throughout the experiment, but increasingly higher correlations between places and words indicated that content became less important during the experiment. Means are controlled for age. Bars denote the SE.

Interestingly, we also found an effect of the scoring method in interaction with repetition and content (familiar/INT). *Post hoc* pairwise *t*-tests (two-tailed) showed that memory for geographic place names was better than for words. This difference stayed significant throughout the experiment, *t_s_* (79) > -4.03, *p_s_* < 0.001, see **Figure 2**. Correlations between memory for place names and words increased when only whole words were scored (Block 1 *r* = 0.51, Block 2 *r* = 0.68, Block 3 *r* = 0.79, Block 4 *r* = 0.79), and also when letter sequences were scored in addition (Block 1 *r* = 0.50, Block 2 *r* = 0.72, Block 3 *r* = 0.84, Block 4 *r* = 0.82), *p_s_* < 0.001. These results suggest that the content of the words became less important for memory performance during the experiment because the shared variance between the two types of memory lists increased. **Figure 2** shows that indeed there is a subtle narrowing of the gap between place names and words during practice which is slightly more pronounced when scoring letter sequences (difference between initial and final gap = 2.51%) than when scoring whole words (difference between initial and final gap = 2.19%).

**FIGURE 3:**
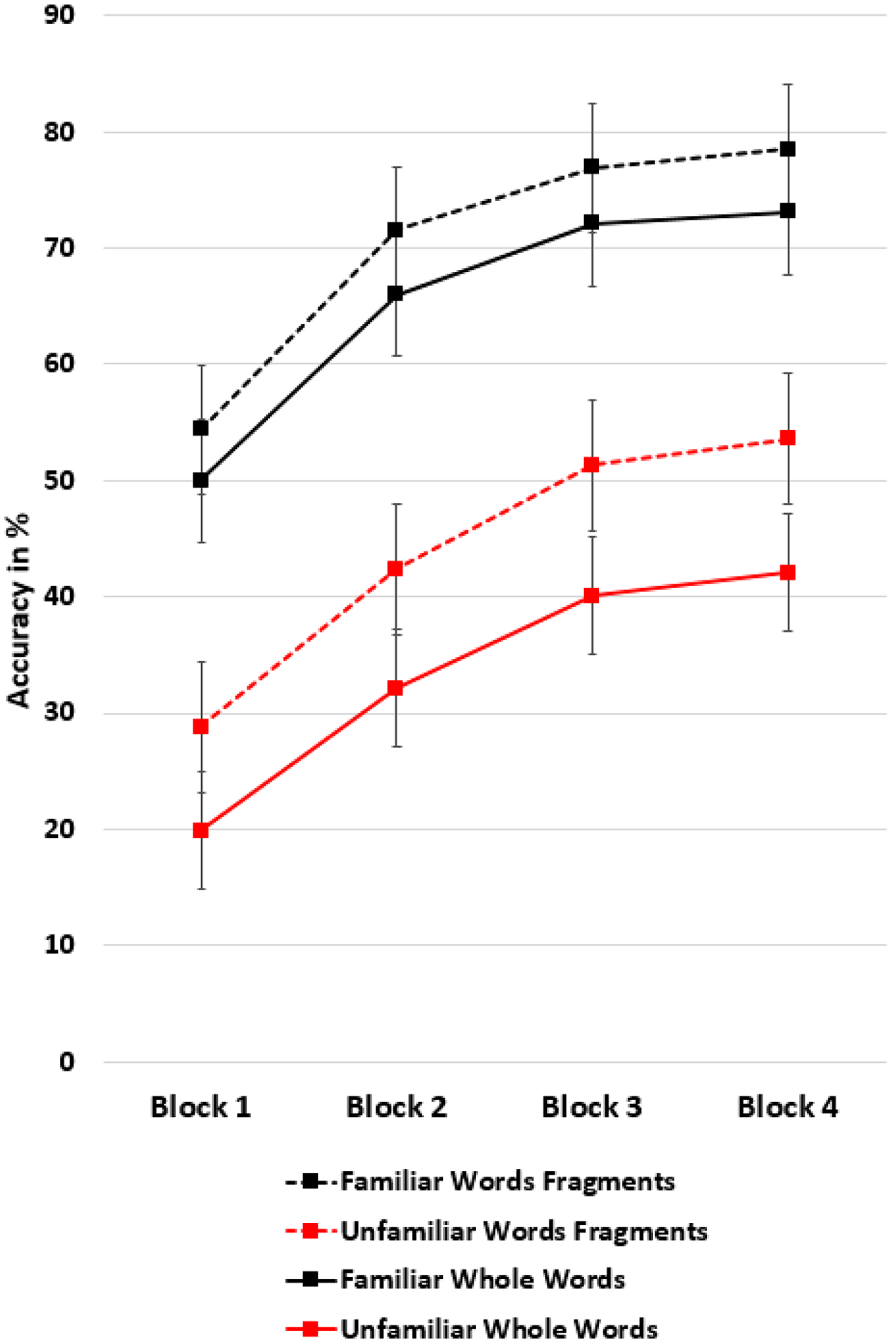
**Effect of familiarity**. It was significantly more likely that unfamiliar words were recalled as word fragments (letter sequences) than familiar words. Means are controlled for age. Bars denote the SE.

Finally, the interaction of scoring by repetition with familiarity showed that participants particularly benefited from the scoring method which appreciated letter sequences when they recalled unfamiliar words as they were significantly more likely to recall unfamiliar than familiar words as word fragments, see **Figure 3**.

### Word Fragments

The second analysis compared the whole word score with the partial word fragment score as described before in the Methods section. Again, recall scores were analyzed with analyses of variance with repeated measures. We conducted again a 2 (Words/Places) × 2 (Familiarity) × 4 (Repetition) × 2 (Scores) × by 2 (Training) analysis of variance with repeated measures on the first four factors, type of training as between-subject factor and age in years as covariate. Also in this second analysis, the statistical effects are listed in **Table 3**, on the right hand side. Most of the statistical effects are the same, however, there are some important differences.

The effect size of the scoring effect more than doubled. This occurred because there were on average significantly fewer word fragments (*M* = 7.7%) than totally correct words (*M* = 57.2%). The scoring effect interacted with familiarity and training. This interaction was further explored with two MANCOVAs (controlled for age) separately for whole words and fragments, respectively. The type of repetition mattered for whole words, *F*(1, 80) = 10.25, *p* = 0.002, h^2^ = 0.12. When familiar words were encountered it made little difference whether they were immediately (*M* = 69.8%) or incidentally (*M* = 70.8%) repeated. In contrast, when unfamiliar words were encountered, these were better remembered when repeated in immediate succession (*M* = 47.5%) than when encountered incidentally (*M* = 40.5%), *t*(78) = 2.26, *p* = 0.027 (two-tailed). The type of training did not matter for word fragments, though: In both memory training conditions, unfamiliar words were more likely to be recalled as a fragment (immediate *M* = 10.8%, incidental *M* = 10.1%) than familiar words (immediate *M* = 5.9%, incidental *M* = 4.2%).

Importantly for the rehearsal hypothesis, the scoring effect interacted with repetition, with a relatively large effect size of η^2^ = 0.44, and this effect did not interact with the timing of the blocks. The amount of orthographically correct recalled words increased with repetition by 24.4% (Block 1 *M* = 41.6%, Block 2 *M* = 56.9%, Block 3 *M* = 64.1%, Block 4 *M* = 66.0%), while the word approximations increased by 1.8% (Block 1 *M* = 6.6%, Block 2 *M* = 7.9%, Block 3 *M* = 8.0%, Block 4 *M* = 8.4%). There was no decrease in word fragments.

Still, there was the possibility that the word fragments did not increase as much because they were feeding into the increase of correctly spelled words. To investigate this question, we computed the four correlations between the four repeated blocks of the whole words and the word fragments, respectively, and the three correlations of word fragments with the subsequent block of whole words. We adapted the level of significance to 0.05/11 correlations = *p* < 0.004. The correlations in **Table 4** show the same correlational pattern for the total sample as for all four sub-samples. The repeated recall of correctly spelled whole words correlated highly and significantly with each other, and likewise, word fragments where only some letters were in the correct sequence correlated significantly with each other from one block to the next, although at a somewhat lower level. However, the recalled word fragments showed not a single significant correlation with correct whole word recall.

**Table 4:** Correlations between whole words and word fragments recall scores across memory blocks.

	Words Block 1	Words Block 2	Words Block 3	Words Block 4	Frgmt Block 2	Frgmt Block 3	Frgmt Block 4
			**Total sample (*n* = 80)**				
Words Block 2	0.87**						
Words Block 3		0.86**					
Words Block 4			0.83**				
Frgmt Block 1		0.10					
Frgmt Block 2			-0.01		0.69**		
Frgmt Block 3				-0.00		0.76**	
Frgmt Block 4							0.75**
			**Monolingual males (*n* = 17)**				
Words Block 2	0.85**						
Words Block 3		0.95**					
Words Block 4			0.97**				
Frgmt Block 1		-0.09					
Frgmt Block 2			0.15		0.74**		
Frgmt Block 3				0.19		0.73**	
Frgmt Block 4							0.83**
			**Bilingual males (*n* = 17)**				
Words Block 2	0.87**						
Words Block 3		0.94**					
Words Block 4			0.92**				
Frgmt Block 1		0.25					
Frgmt Block 2			-0.24		0.68*		
Frgmt Block 3				-0.06		0.81**	
Frgmt Block 4							0.70*
			**Monolingual females (*n* = 20)**				
Words Block 2	0.88**						
Words Block 3		0.92**					
Words Block 4			0.94**				
Frgmt Block 1		-0.34					
Frgmt Block 2			-0.15		0.83**		
Frgmt Block 3				0.19		0.84**	
Frgmt Block 4							0.73**
			**Bilingual females (*n* = 26)**				
Words Block 2	0.87**						
Words Block 3		0.94**					
Words Block 4			0.95**				
Frgmt Block 1		0.29					
Frgmt Block 2			-0.05		0.59*		
Frgmt Block 3				-0.13		0.71**	
Frgmt Block 4							0.75**

Frgmt = Fragments; **p < 0.001; *p < 0.004.

## Discussion

Working memory as well as psycholinguistic research usually assumes that rehearsal is based on the phonological loop (Gathercole and Baddeley, 1993, 1997; Edwards et al., 2004; Gupta and Tisdale, 2009). In particular, the processing of non-words gives important cues to language learning (Gathercole, 2006a,b). We do not doubt these findings, but we do doubt that the phonological loop and (sub-vocal) articulation are the only relevant systems of word memory. Page et al. (2006, p. 732), for instance, write that when access to the loop would be blocked by concurrent articulation, participants would need to fall back ‘on a largely unrehearsable visual store.’ Importantly for the current study, Page and Norris (2009) assume that the repetition and rehearsal of a word actually builds a long-lasting long-term memory (LTM) representation, but of a phonological word-form in the mental lexicon. However, Darling and Havelka (2010) and Darling et al. (2012) suggested instead that visual and verbal information of words are bound together in the multi-sensory episodic memory system which is integrated into the working memory system (Logie, 2011; Baddeley, 2012). Likewise, in many word recognition models in reading research, grapheme–phoneme correspondences are assumed to be made when reading aloud (Coltheart, 2012).

In the current study, participants were writing down responses in a free word recall task, and hence they had to resort to visual orthographic patterns that they saw before. They saw them several times which gave them the opportunity to improve their memory performance. However, participants did not only reproduce the actual words from the memory list, but also wrote down partially correct words. These sublexical word structures were found to occur also in spoken responses (Storkel et al., 2006). They are also common in children, for instance, Treiman (1993) showed that first graders’ correct spellings increased within 1 year from 888 to 1,989 correct spellings (124%), but so did the wrong spellings from 1,135 to 1,605 wrong spellings (41.4%). Thus, both accurately spelled words and words with wrong spellings increased, albeit at different rates. Also in the current experiment with young adults, however, written word fragments increased during repetition and this showed no trade-off with correct words. The word fragments were learned insofar as scores were correlated with each other during repeated blocks, but not with correct words. This kind of *error learning* during repetition also occurred in a serial recall task using just letters (Couture et al., 2008) and in a word memory task (Storkel et al., 2006). In short, the current study makes a case that word fragment learning showed the two sides of rehearsal and repetition: not just accurate responses, also the probability of giving a wrong response increases with the number of prior occurrences of that response.

### Visual Orthographic Patterns of Letters

Friederici and Lachmann (2002) came to the conclusion that there are no brain areas which are originally reserved for reading words. In development, brain areas with other primary functions such as syntactic processing when reading sentences, face recognition in the case of visual complex pattern recognition when identifying words, or the lexicon for spoken words when matching phonological forms, are recruited for reading print. The most direct way to encode in a visual word memory task where words are read from the screen and written down during recall would be *visual mapping* (Lange-Küttner, 2014) or *visual bootstrapping* (Darling et al., 2012).

However, modalities can or should interact in word memory. Page et al. (2006) investigated the effects of repetition in the two modalities. Adults’ learning effects during repetition were locked into one modality without any transfer in the case of letters and pictures. However, when words were used, transfer occurred in the visual-then-auditory condition, but not in the auditory-then-visual condition: A sound was associated with a visual word seen before, but a visual word was not associated with a spoken word heard before. Hence, we may be more likely to enliven the ‘graphic imagery’ of a written word with a sound than to think about how a word is written after hearing it. This seems to indicate that we do not have much visual imagery for written words.

Also in children, there was a clear culturally and educationally shaped preference of children to recruit one modality only for reading, either visual or auditory word memory (Lange-Küttner and Krappmann, 2011). This selectivity in memory has been emphasized since quite some time (Cowan, 1995). In fact, when neural networks were run, double-modality word input and double encoding was most beneficial for immediate word reproduction, but only one working memory system was necessary to integrate a letter sequence for word learning to occur (Lange-Küttner and Krappmann, 2011). Boys seem to have a preference for the visual modality which includes perception of the fine visual detail of the orthographic letter patterns (Mohamed et al., 2011; Huestegge et al., 2012). These orthographic patterns are assumed to be stored in the brain in the ‘visual word form system’ and can be evoked by writing (Dehaene et al., 2010). The letters in the orthographic pattern allow a much more precise notation of sounds than is apparent in the sonograms of spoken language where not only individual words but also individual sounds present a segmentation problem (Whitney, 1998, p. 142; Lange-Küttner et al., 2013). The digital transformation of naturally spoken speech into written words still represents a major challenge for typing software. Likewise, in school children the best predictor for writing inner speech into a fluent text – besides writing speed – is word spelling accuracy (Connelly et al., 2012). Thus, one would suppose that also visual orthographic patterns of letters are important for verbal memory and can be rehearsed.

We found indeed that in the written responses of our participants, mere repetition nearly doubled accuracy which clearly supports the hypothesis that prescribed rehearsal is an efficient facilitator for word memory. For familiar words it made little difference whether they were immediately or incidentally repeated, while the unfamiliar INT words were better remembered when repeated in immediate succession. The unfamiliar INT words were also more likely to be recalled as a fragment than familiar words. This suggests that unfamiliar words with foreign spellings benefit from immediate rehearsal that builds up a visual orthographic template in LTM within a relatively short time. Writing an unfamiliar word correctly, however, is a fragile process which was not helped by immediate repetition. In the following section we discuss why this may be the case.

### Error Learning in Word Memory during Repetitions

We paid particular attention to the orthography that the participants produced when recalling the word lists and writing down their responses. When words were not spelled correctly yet still identifiable as memory of the correct word, we scored the letters in the right sequence as per cent of the actual word length. We wanted to know whether these letter sequence word fragments would develop into proper whole words if rehearsed several times. We predicted that if this would be the case, word fragments should decrease during the repetition, while the whole word score should increase. This hypothesis was partly confirmed. One the one hand, it was true that rehearsal in the repeated memory blocks produced a higher whole word memory score, on the other hand, word fragments did not decrease, but increased too. Thus, the expected trade-off between word fragments and whole words did not occur. Instead, also the word fragments increased with repetition, albeit by a smaller amount, but then there were also fewer word fragments than whole words in participants’ response sheets. Word fragments were more often produced in response to unfamiliar words, e.g., in response to the INT non-words with legal letter combinations from other languages and INT geographical places also following non-English language spelling rules.

This confirmed results from a developmental study with 8- to 10-year-old children showing that non-words created from the native language were easier to learn than non-words created from a non-native language (Morra and Camba, 2009). The increase of word fragments suggests that during the experiment, participants kept trying to cobble together letters into word patterns that resembled the visual input word to some degree, not unlike the 5-year-old reading beginners adept in distinguishing visual word fragments (Rack et al., 1994).

Orthographic patterns were also scored in a serial recall task of letters with adults (Couture et al., 2008). Also in this study, correct recall of a letter in the right place showed the same learning curve during repetition as erroneously recalled letters, that is error learning occurred. Interestingly, in the study of Couture et al. (2008) the error learning during repetition occurred only when data of real people were analyzed, but not when simulated data were used which yielded an increase in correct answers while wrong responses stayed at floor level.

In another study also partially correct responses were analyzed, but stimuli and responses were spoken (Storkel et al., 2006). The auditory format enabled the authors to control the stimuli for phoneme transition difficulty (ease of pronunciation) and lexical neighborhoods (number of similar words), two factors which impact on non-word learning quite independently of each other (Bailey and Hahn, 2001). Storkel et al. (2006) showed that scores of both completely correct words and partially correct sublexical word units increased during repetition. This repetition effect did neither interact with lexical neighborhood density nor with phonotactic probability of the words. Correct words increased at a steady rate throughout seven repetitions, while partially correct words leveled off after four exposures. However, no statistical comparison was made which would have shown whether this difference would have amounted to a significant interaction that denoted a trade-off between partially correct words and complete words. Thus, in this study it remains unclear whether adults could transform a spoken word approximation into a proper word during repetition. To our knowledge, only two studies so far showed that errors were actually decreasing during repetition. One study used 10-item digit sequences from 0 to 9 in an immediate serial recall task (Cumming et al., 2003). Importantly, errors were omission mistakes where participants would initially fill in blanks, but during repetition became able to fill the gaps. The same effect of repetition was found when letter sequences were used (Couture and Tremblay, 2006, Experiment 3). However, this was not the case in 5–6 years old children who improved with repetition, but not by supplementing missing information in serial positions (Mosse and Jarrold, 2010). This may have been the case because at this age, children are not yet fluent readers, and when they spell words with letters, commission errors are more frequent than omission errors or reversals in the letter sequence (Treiman, 1993). Nevertheless, the 5–6 years old children’s learning during repetition correlated significantly with learning non-words, but not with regular word learning. Hence, one conclusion could be that the input repetition effect seems to transform novel information into familiar information that can potentially be incorporated into a systematic database (Plunkett and Marchman, 1990).

In the current study, it is very likely that the correctly written down whole words were rehearsed via inner speech which speaks to a straightforward involvement of the lexicon and semantic LTM. Also in the Storkel et al. (2006) study, memory for complete spoken words was determined by lexical neighborhood density only.

However, in the current study it is less likely that also the partly correct written down word fragments were processed via lexical access because they were immune to content and presentation distribution effects. The amount of word fragments occurred also independently of individual differences with regards to sex and language. We must assume that when word fragments were written down as a response that this visual orthographic pattern was remembered from the presentation. A fragmented visual registering of the word input may be responsible for partial recall because inserting a delay before a recall test which could have been used for enhanced recovery did not make any learning difference (Oberauer and Meyer, 2009). The repeated learning would then serve as a kind of sensory visual learning (Mortensen and Nachtigall, 2000; Blum and Yonelinas, 2001) until an accurate word form has been registered that can be associated with some meaning. Also in the Storkel et al. (2006) study with spoken word stimuli, the partially correct words were not lexically retrieved, but instead phoneme transitions of the words were important. Hence, one could conclude that learning of novel unfamiliar words can begin on a very raw sensory level, for spoken words with acoustic sounds and for written words with graphemes.

This result of different processes for complete vs. partial word memory during rehearsal and repetition is further underpinned by the finding that the increase in complete words and the increase in word fragments occurred independently of each other, as we could not find significant correlations between them. Word fragments in the repetition were highly and significantly correlated with each other in the total sample, with *r* values between 0.69 and 0.75. This was somewhat lower than for whole words which correlated very highly between 0.83 and 0.87 in the repetitions. This correlational pattern could be replicated with a split-file method, with *r* values between 0.59 and 0.83 for word fragments and *r* values for whole words between 0.85 and 0.95 in the repetitions. We tested hypothesis-guided planned correlations and predicted that the word fragment score in one block would correlate with the whole word score in the next Block. However, these and also almost all of the other correlations between word fragment scores and subsequent whole words scores were not significant.

Moreover, we would like to suggest that it is likely that also the increase of word fragments consisted of two processes. The first process would be the rehearsal of the word fragment, and this explains why there were significant correlations that could increase during the repetition. The second process would be that increasingly some more new word fragments were produced, and this relatively free generative process explains why the correlations were on average lower than for whole words. In the context of an immediate serial recall task, Couture et al. (2008) found that repeated learning of visual letter sequences yielded 2,376 response mouse clicks. Of these clicks 938 responses were errors, with 468 repeated errors and 470 new errors. 159 repeated errors were from the previous block, but 309 errors were from an even earlier block in the experiment. This indicates that wrong letter sequences were well remembered in visual LTM beyond the immediate recall context. When increasing error learning during repetition is not analyzed this could be mistaken for an absence of correct response learning, while in fact both correct and wrong responses increase simultaneously (Lafond et al., 2010). Also McClelland (2001) warned that Hebbian learning may actually strengthen inappropriate activations if for instance an over-inclusive prototype was generated during learning.

### Sparse Written Word Representations

Would partially correct words be similar to Mojibake? A Mojibake of unintelligible characters emerges when different writing systems clash, such as Japanese Kanji JIS and the Western Alphabetic code ASCII (Wlodarczyk, 2005). It is even suggested to make PDF word documents safer by using Mojibake (Bakhtiyari et al., 2014). PDF documents have an upper layer with an image of the text and a lower layer with the letters that make up the words. It is suggested that a way to increase PDF security would be to eliminate the letter sequences and instead of well-sequenced letters only Mojibake would be offered in the lower layer which would render copying of the PDF document impossible.

This suggests that there may be also two layers of word memory in participants, and not just in PDF documents. The current experiment showed that there may be a lower sensory layer consisting of free sparse word fragments which can be image-like pictures or acoustic-like sounds and an upper layer that consists of language-specific, orthographically and semantically constrained words. This is just the opposite of what was suggested by Chomsky for spoken language (Chomsky, 1959, 2002; Chomsky and DiNozzi, 1972). He suggested that we are creative rather than conditioned insofar as there is a lower layer of deep meaning anchored in action schemata, while the human mind finds myriads of ways to express the meaning in syntactic structures on a surface level. However, the current study shows that when top–down word representations from a mental lexicon cannot trigger an unfamiliar word from the LTM store because of complete novelty, or a small constrained lexicon, incomplete sparse sublexical bottom-up sensory impressions of word input take over (see also Nuerk et al., 2000) which are reinforced over repetitions even if partly wrong.

A similar explanation was given by Frick (1988) who wrote an immensely instructive early review about learning with repetition, in particular the Hebb effect. The Hebb effect shows that dispersed repeated sequences of letters, digits or words are better learned than novel sequences in an immediate recall task, even if participants do not notice the repetition (see also McKelvie, 1987). Frick suggested a *recorder model* with a fixed amount of recording tape. Thus, in general, reproduction of words would show high fidelity of the original word. However, when too many items are presented, only a small amount of representational medium could be devoted to each item resulting in a low fidelity representation. He described that while participants represent a set of words, they do not represent psychophysical parameters such as duration, or mimic the pitch, accent, rhythm or loudness (Frick, 1988, p. 223, but see Lange-Küttner et al., 2013). Instead, an unparsed, uncategorized, more or less degraded input would need to be recovered for recall. According to Frick, the recovery for recall would represent a second level of processing which can be facilitated with grouping or chunking (see also Cumming et al., 2003) into categories or perceptual boundaries of Gestalt-like stimuli and stimulus sequences.

We would suggest that in the case of written words, this process of recovery is not creative but on the contrary, it is conventional insofar as it is governed via the lexicon that prescribes an exact replication of the graphic orthography. In terms of working memory, the inner scribe and the visual cache components of the visual-spatial sketchpad of the working memory model may be likely candidates for the visual rehearsal of words fragments. Logie (2011, p. 214) describes visual rehearsal as follows: ‘The Inner Scribe component (…) can allow visual codes to be held for longer by mentally rehearsing the codes held in the Visual Cache.’ Thus, we would suggest that rehearsal of written word fragments is most likely to take place in the inner scribe and the visual cache, firstly because participants held some sparse details of recently perceived unfamiliar words (in the visual cache), and secondly, during the repetition these were processed further (in the inner scribe). However, in order to avoid learning wrong words, an active mapping process would need to take place where the visual slave systems are controlled by the central executive whether the visual orthographic code matches LTM representations in the episodic memory system that stores accumulated conventional orthographic patterns encountered during previous experiences.

Storkel and Rogers (2000) showed that in spoken language, children were drawing an advantage from more easily pronounceable words in word recognition only from age 10. This late onset suggests that in word memory children develop language-specific acoustic and probably also graphonomic sensitivities relatively late after being taught to read. It also suggests that increased sensitivities may need an increased categorical filter or quality control. For instance, children seem to be biased toward positive feedback whether it is justified or not (Crone et al., 2004; Eppinger et al., 2009; Lange-Küttner et al., 2012) which may help to persevere in a learning task, but not to discriminate when words do not ‘look right.’ Moreover, the current study showed that this is still the case in young adults if they encounter unfamiliar words with no ready-made word template available for word recall.

### Future Research Questions

In development, the onset of written language changes word memory because the new visual modality is added to language. For instance, in beginning readers, their small lexicon of written words makes them rely heavily on familiar items in their visual word memory, while the saturated lexicon of spoken words accumulated over several years allows them to better memorize novel words (Lange-Küttner and Martin, 1999; Lange-Küttner, 2005; Lange-Küttner and Krappmann, 2011). Also children with reading difficulties produce significantly more misspellings that are close visual matches to the target word rather than phonological mismatches (Lennox and Siegel, 1996). This is why the current study put more weight on orthographic patterns in visual word memory than on phonemic sound transitions in spoken word memory. Visual word rehearsal may be counter-intuitive, but for written language it is quite a crucial research question that needs further testing. For instance, while it is a reasonable assumption that word fragments develop into whole words, the current study did not find any statistical evidence for a trade-off between word fragments and whole words.

The finding of a persistent proportion of word fragments in free recall is rather worrying. It has indeed been claimed recently that error learning during repetition may be responsible for developmental dyslexia (Szmalec et al., 2011). While learning with repetition was completely absent in dyslexic participants when they had to remember the places of dots, it was only attenuated in visual and auditory learning of letter sequences. Likewise, also children with Down syndrome showed learning with repetition comparable to normally developing children which explained their good vocabulary despite a verbal short-term memory deficit (Mosse and Jarrold, 2010).

Although the current study could not show that word fragments would develop into a whole words during repeated rehearsals, there is a hint in the non-significant correlations, which developed from a negative into a positive correlation (monolingual males and females), or from a positive into a negative correlation (bilingual males and females) during the experiment, see **Table 4**. While this appeared to be a smooth trend, none of these correlations ever reached significance. We also tried to increase the correlations by distinguishing between word fragments in response to familiar vs. unfamiliar words, but again without obtaining significant correlations with whole word responses of the same kind.

The comparison with previous research showed some indicators that rehearsal of whole words and word fragments is based on two different cognitive processes. Future research could use an item-based methodology where the fate of an individual word fragment is followed up. For instance, Carey (2010) assumes that extended mapping with context information produces more constrained meaning in words that were acquired via fast word mapping. Hence, extended mapping could facilitate the transition of a response from the lower free sensory layer to the upper semantically and orthographically constrained layer. This transformation from a word fragment to a proper word recall could be tested using the category size effect (Hunt and Seta, 1984). This effect demonstrates that words from small categories are better recalled following orientating relational processing, and words from large categories are better recalled following individual item processing. One could envisage an experiment where an increasingly longer word list in the repetitions gradually provides more context which could support the refinement of a word fragment into a correct whole word, or an experiment where a word list gradually becomes more homogeneous during repetition. For example, if the Word List with EU towns would gradually change into a Word List with French towns only, providing a more systematic database, would the first initially introduced French town that was recalled as a word fragment be spelled correctly once all town names are presented in the same language? In this item-based experiment, unbeknown to the participants, only the rehearsal of the first word fragment would be important, while the remaining words could be left unscored.

We conclude that the current study provided compelling evidence that written word fragments are likely to be produced when unfamiliar words are encountered, and that these word fragments are rehearsed and increase during repetition. We suggest that written word fragments seem to be free and highly idiosyncratic which currently makes it difficult to demonstrate how a written word fragment can be rehearsed until a whole word emerges. We suggest that extended mapping may simultaneously constrain the semantic content and the orthography of a written word fragment so that it ‘looks right.’

However, it is also imaginable that word fragments never develop into proper words but persist in memory. In the development of young children’s first spoken word production, invented words were found to be abruptly dropped in favor of conventional words only (Dromi, 1987). Anecdotal evidence from children shows that strict rules can control orthographic output and inhibit the rehearsal activity at the lower level rather than evolve it. We introduced this study with the neural network simulation of the Ebbinghaus study (Lange-Küttner, 2011) because Ebbinghaus (1964) learned the nonsense syllables always to perfection and the gains that he described were only in terms of time. However, a focus on perfect accuracy may inevitably simultaneously inhibit the learning potential with regards to memory for unfamiliar words of any kind. Hence, to investigate error learning and the interactivity between fragile letter sequences and robust word representations is an important future research goal.

## Conflict of Interest Statement

The authors declare that the research was conducted in the absence of any commercial or financial relationships that could be construed as a potential conflict of interest.
